# Implantation of atrial flow regulator devices in patients with congenital heart disease and children with severe pulmonary hypertension or cardiomyopathy—an international multicenter case series

**DOI:** 10.3389/fcvm.2023.1332395

**Published:** 2024-01-15

**Authors:** Gianfranco Butera, Enrico Piccinelli, Adam Kolesnik, Konstantin Averin, Cameron Seaman, Biagio Castaldi, Elena Cuppini, Alain Fraisse, Carles Bautista-Rodriguez, Sebastien Hascoet, Carmen D'Amore, Alban-Elouen Baruteau, Pedro Betrián Blasco, Lisa Bianco, Andreas Eicken, Matthew Jones, James A. Kuo, Grazyna Brzezinska Rajszys

**Affiliations:** ^1^ Cardiology, Cardiac Surgery and Heart Lung Transplantation, ERN GUARD HEART, Bambino Gesù Hospital and Research Institute, IRCCS, Rome, Italy; ^2^ Department of Mechanical and Aerospace Engineering, Politecnico di Torino, Torino, Italy; ^3^ Cardiovascular Interventions Laboratory, The Children’s Memorial Health Institute, Varsavia, Poland; ^4^ Division of Cardiology, Department of Pediatrics, Stollery Children’s Hospital, Edmonton, AB, Canada; ^5^ Pediatric Cardiology Department, Padova University, Padova, Italy; ^6^ Pediatric Cardiology Department, Royal Brompton Hospital, London, United Kingdom; ^7^ Epidemiology, Clinical Pathways and Clinical Risk Unit, Medical Direction, Bambino Gesù Children’s Hospital, IRCCS, Rome, Italy; ^8^ Nantes Université, CHU Nantes, Department of Pediatric Cardiology and Pediatric Cardiac Surgery, FHU PreciCare, Nantes, France; ^9^ Pediatric Interventional Cardiology Unit, Vall d'Hebron Hospital, Barcellona, Spain; ^10^ Department of Congenital Heart Disease and Pediatric Cardiology, Deutsches Herzzentrum München, München, Germany; ^11^ Department of Paediatric Cardiology and Adults with Congenital Heart Diseases, Evelina Children’s Hospital, London, United Kingdom; ^12^ Division of Cardiology, Children’s Healthcare of Atlanta, Atlanta, GA, United States

**Keywords:** congenital heart disease, atrial flow regulator device, Fontan circulation, pulmonary hypertension, extracorporeal membrane oxygenation

## Abstract

**Background:**

The Occlutech Atrial Flow Regulator (AFR) is a self-expandable double-disc nitinol device with a central fenestration. Its use has been approved in the adult population with heart failure and described for pulmonary hypertension (PH). Only case reports and small series have been published about its use in the paediatric population and for congenital heart disease (CHD).

**Objectives:**

The authors sought to investigate the feasibility, safety, and short-term follow-up of AFR implantation in patients with CHD or children with PH or cardiomyopathy.

**Methods:**

This is a multicenter retrospective study involving 10 centers worldwide. Patients of any age with CHD or patients aged < 18 years with PH or cardiomyopathy needing AFR implantation were included.

**Results:**

A total of 40 patients underwent AFR implantation. The median age of the population at the time of the procedure was 58.5 months (IQR: 31.5–142.5) and the median weight was 17 kg (IQR: 10–46). A total of 26 (65.0%) patients had CHD, nine (22.5%) children, a cardiomyopathy, and five (12.5%), a structurally normal heart. The implantation success rate was 100%. There were two early and one late device thrombosis. Two patients (5.0%) with dilated cardiomyopathy on extracorporeal membrane oxygenator (ECMO) died during the hospital stay. At a median follow-up of 330 days (IQR: 125–593), 37 (92.5%) patients were alive. At follow-up, 20 patients improved their New York Heart Association (NYHA) class, 12 patients did not change their NYHA class, and one patient with idiopathic PH worsened.

**Conclusions:**

AFR implantation in patients with CHD and children with severe PH or cardiomyopathy is promising and seems to have beneficial effects at short-term follow-up.

## Introduction

The Occlutech Atrial Flow Regulator (AFR) (Occlutech Holding AG, Schaffhausen, Switzerland) is a self-expandable double-disc nitinol device with a central fenestration, approved in Europe only for patients with heart failure with preserved and reduced ejection fraction (1, 2) and currently for compassionate use in patients with severe pulmonary hypertension (3–5). It is available in 4, 6, 8, and 10 mm but only 8- and 10-mm devices have a CE Mark in adult patients with heart failure with preserved or reduced ejection fraction. Its off-label use with internal technical review by Occlutech has been described in patients with congenital heart disease (CHD), especially in children with Fontan palliation to create or reduce the size of the conduit fenestration, but also in patients with cardiomyopathy (6–15). Finally, it has been successfully implanted in children on extracorporeal membrane oxygenator (ECMO) to unload the left cavities (16, 17).

Despite the enormous potential of this device in many congenital settings, the current literature is anecdotal and large series and long-term multicentric trials are missing. The aim of this study is to describe the clinical characteristics and the short-term follow-up of patients with CHD and children with severe right heart failure due to pulmonary hypertension or cardiomyopathy necessitating the implantation of an AFR.

## Material and method

This multicenter study was conducted in 10 international tertiary care pediatric cardiology centers. All consecutive patients at any age with CHD or patients aged less than 18 years with pulmonary hypertension or cardiomyopathy needing AFR implantation between June 2017 and September 2022 were retrospectively included. The indication for AFR implantation was decided independently by each cardiac center within their respective multidisciplinary team. The procedure was performed by at least two expert congenital interventional cardiologists. Demographical and clinical information was collected. The indications for AFR implantation were divided into five groups: left heart failure, right ventricle hypertension, severe desaturation in fenestrated Fontan, Fontan failure, and to unload left cavities during the ECMO run. Procedural and postprocedural data, including details on the technique, device size, complications, postoperative medications, and intensive care stay were also recorded. Finally, data regarding the last follow-up such as clinical status, device patency, and late complications were collected. The New York Heart Association (NYHA) Functional Classification was used to assess the functional status of the patients before and after the procedure. The institutional review committee approved the study and all patients or their caregivers gave their informed consent for the procedure.

## Statistical analysis

Patients were described according to their demographical and clinical characteristics. Collected data were presented as count and proportions (categorical data) or median and interquartile range (continuous data). Comparisons between groups were performed using Chi-squared test for categorical data while Mann–Whitney *U* test and Kruskal–Wallis test were used for continuous data. A *p*-value < 0.05 was considered statistically significant. All statistical analyses were conducted using STATA 17 (Stata Corporation, College Station, Texas, USA).

## Results

### Demographic and clinical characteristics of patients

A total of 40 patients were included in this international study. There were 33 pediatric patients and seven adult patients. The median age of the population at the time of the procedure was 58.5 months (IQR: 31.5–142.5 months) and the median weight was 17 kg (IQR: 10–46 kg). According to the baseline anatomy, 26 patients had congenital heart disease, nine patients had cardiomyopathy, and five had a structurally normal heart. General characteristics and indications for AFR implantation are reported in Table 1. Approximately 55% of all patients had at least one previous surgical or percutaneous intervention: nine patients with single ventricle physiology were palliated with Fontan circulation, nine patients reached a biventricular repair, and four needed ECMO because of end-stage cardiomyopathy (Table 1).

**Table 1:** Demographic and clinical characteristics of the study population at the baseline.

Variable	Number of patients (*N* = 40)	%
Gender		
Male	25	62.5
Female	15	37.5
Baseline anatomy		
Congenital heart disease	26	65.0
Left heart obstruction	11	42.3
Hypoplastic left heart syndrome	8	30.7
Secundum atrial septal defect	4	15.4
Right heart obstruction	2	7.7
Hemi-truncus arteriosus	1	3.9
Cardiomiopathy	9	22.5
Dilated	6	66.7
Restrictive	2	22.2
Hypertrophic	1	11.1
Structurally normal heart with idiopathic pulmonary hypertension	5	12.5
Indication for AFR implantation		
Left heart failure	16	40.0
Right ventricle hypertension	11	27.5
Fenestrated Fontan desaturation	5	12.5
Fontan failure	4	10.0
ECMO	4	10.0
Baseline NYHA class		
I	2	5.0
II	13	32.5
III	17	42.5
IV	7	17.5
Missing	1	2.5

### Procedural data and early results

An AFR device was successfully implanted in all patients with a median procedure time of 81.5 min (IQR: 41–135) and a median radiation exposure of 715.0 cGy/cm^2^ (IQR: 180–1,877). Almost all the procedures (95.0%) were performed under general anesthesia, while 5.0% were done under conscious sedation. Venous access was femoral in 36 patients (90.0%), transhepatic in 2 patients (5.0%), subclavian in one patient (2.5%), and hybrid transatrial in one patient (2.5%). Heparin was administered at the beginning of the procedure or after transeptal puncture at an initial dose of 100 IU/kg with an activated clotting time (ACT) target >200 s. Periprocedural antibiotic prophylaxis was administered to all patients. Baseline hemodynamic data were obtained (Table 2). The maximum venous sheath was 16 Fr (range 8–16F). Access to the left atrium was obtained with a transeptal needle in 25 patients (22 interatrial septa and three Fontan conduits), in nine cases the device was implanted through an existent interatrial communication and in six cases through a Fontan fenestration (the five patients with severe desaturation in a fenestrated Fontan and one patient with a failing Fontan with a small fenestration). The fenestration was then dilated with a balloon before AFR implantation in 26 patients (65.0%). The most used balloon was the Powerflex PTA balloon (Cordis corporation), which was used in nine patients, followed by cutting balloons in five patients, the Sterling PTA balloon (Boston Scientific), Advance PTA balloon (Cook Medical), and Atlas Gold PTA balloon (Bard Medical) in three patients, and Conquest or Dorado PTA balloons (Bard Medical) in two patients. More than one different balloon was needed in six patients. The mean balloon size: AFR Fenestration ratio was 1:6. The most used device was the 4-mm one in 13 patients (32.5%), followed by the 8 mm in 12 patients (30.0%), the 6 mm in nine patients (22.5%), and the 10 mm in six patients (15.0%). The median gradient across the AFR device at the end of the procedure was 8.5 mmHg (IQR: 4–12 mmHg) with mainly left-to-right shunt in patients with left heart failure or ECMO and right-to-left shunt in patients with Fontan circulation or right ventricle hypertension. There was a 5.0% rate of intra-procedural complications, consisting of two immediate AFR occlusions. The first was a patient weighing 13.9 kg with a Fontan failure in which a 4-mm AFR was used to decompress the circulation. The device clotted soon after implantation during the procedure despite heparin, and it was left in place and replaced with a 6-mm device 1 month later. In the second patient weighing 19.6 kg with a fenestrated Fontan with desaturation, a 4-mm device clotted soon after implantation during the procedure despite heparin. It was removed, snaring the device, and replaced with a 6-mm device during the same procedure. No intraprocedural deaths were registered.

**Table 2:** Baseline hemodynamic data.

Indication for AFR implantation	Right atrium mean pressure (mmHg)	Left atrium mean pressure (mmHg)	Systemic ventricle end-diastolic pressure (mmHg)	Pulmonary artery mean pressure (mmHg)	Fontan conduit mean pressure (mmHg)
Left heart failure	12.5 (9.25–13.5)	22 (18.25–28.5)	22 (18.5–22.25)	42.5 (28.9–52.5)	–
Right ventricle hypertension	12 (6–14)	12.5 (8–14.5)	10	35 (31.75–41.5)	–
Fenestrated Fontan desaturation	–	7 (7–9)	9.5 (7.5–11.5)	13 (10.75–14.25)	13 (11.5–14.25)
Fontan failure	–	8.5 (6.5–10)	9 (6–10.5)	16.5 (14–18.5)	16.5 (14.5–18.5)
ECMO	6 (3.75–6.75)	16 (7.5–32)	–	–	–

### Postprocedure data

About 65% of the patients were admitted to intensive care unit (ICU) after the procedure with a median length of ICU stay of 5 days (IQR: 1–15). The median length of hospital stay was 5 days (IQR: 1–20). Two children (5.0%) died during the hospital stay and one infant experienced a severe complication not directly related to the implanted device. More in detail, an 8-year-old child affected by dilated cardiomyopathy on ECMO died of sepsis 16 days after the implantation of a 10-mm AFR to unload the left cavities on ECMO. Similarly, a 10-year-old child affected by dilated cardiomyopathy died of severe brain hemorrhage on ECMO 24 days after the implantation of a 10 mm AFR to unload the left cavities on ECMO. Finally, a 7-month-old patient with dilated cardiomyopathy survived a cardiac arrest and cerebral ischemia few hours after a hybrid procedure of pulmonary artery banding and AFR implantation, needing an ECMO run for 5 days. In all these three patients, the device remained patent.

A total of 38 patients were discharged alive with a patent AFR device. Anticoagulant or antiplatelets therapy at discharge was hugely variable. Indeed, 32.5% were discharged on Warfarin, while novel oral anticoagulant drugs (NOACs) were used in three patients without complications. Another 25.0% of patients were discharged on double antiplatelets (aspirin and clopidogrel) and the remaining with single antiplatelets (aspirin). None of the patients were administered clopidogrel loading before the procedure, aspirin was continued for at least 6 months.

### Follow-up data

At a median follow-up of 330 days (IQR: 125–593), 37 (97.4%) of the discharged patients were alive without experiencing any complications. Only a 4-year-old child with Hypoplastic left heart syndrome (HLHS) died of sepsis in the local hospital 103 days after the implantation of a 6-mm AFR implanted for Fontan failure. During the follow-up, three patients (two cardiomyopathies on ECMO and one critical aortic stenosis with biventricular restrictive physiology after multiple surgical interventions) successfully underwent heart transplantation and one patient underwent ventricular assist device (VAD) implantation. All devices maintained their patency on echocardiography at the follow-up with the exclusion of a 4-mm AFR which clotted 14 months after implantation in a 10-kg child with a Shone-like physiology, despite therapy with aspirin and clopidogrel. It was successfully treated with balloon angioplasty.

Thus, in total there were two early and one late thrombosis on 40 implanted devices, which represented 7.5% of all devices implanted. None of these patients experienced a stroke or died as a result of device thrombosis. Patients with device thrombosis did not appear to differ significantly from those without thrombosis in terms of age (median age 52 months, IQR: 30–58 vs. 60 months, IQR: 33–153; *p* = 0.4) and weight (median weight 14.0 kg, IQR: 10–20 vs. 17.5 kg, IQR: 10–47; *p* = 0.6). At the bivariate analysis there was no difference in AFR device thrombosis between patients with congenital heart disease or not (*p* = 0.2) and according to the NYHA class at presentation *p* = 0.8. The smallest 4-mm device clotted significantly more than the group constituted by 6-, 8-, and 10-mm devices (*p* = 0.009).

Interestingly, only 4-mm devices clotted despite anticoagulant or antiplatelets therapy, which represented 23.1% of this size of devices implanted. Finally, the device clotted in two out of nine patients (22.2%) with Fontan circulation, which was not statistically significant if compared with patients with other indications (*p* = 0.06). At the bivariate analysis, there was no significative difference in the survival outcome between patients with CHD and patients without (*p* = 0.3). AFR implantation in patients with ECMO was significantly associated with death in comparison to other indications (*p* = 0.01) (Tables 3, 4).

**Table 3:** Characteristics of AFR device thrombosis.

	Patients with thrombosis (*N* = 3)	Patients without thrombosis (*N* = 37)	*p*-Value
Baseline anatomy			
Congenital heart disease	3 (100.0)	23 (62.2)	*0*.*2*
Non-congenital heart disease	0 (0.0)	14 (37.8)	
Baseline NYHA class			
I–II	1 (33.3)	14 (37.8)	*0*.*8*
III–IV	2 (66.7)	22 (59.4)	
Missing	0 (0.0)	1 (2.7)	
AFR used			
=4 mm	3 (100.0)	10 (27.0)	*0*.*009*
>4 mm	0 (0.0)	27 (73.0)	
Indication for AFR implantation			
ECMO	0 (0.0)	4 (10.8)	*0*.*5*
Fenestrated Fontan desaturation	1 (33.3)	4 (10.8)	
Fontan failure	1 (33.3)	3 (8.1)	
Right ventricle hypertension	0 (0.0)	11 (29.7)	
Left heart failure	1 (33.3)	15 (40.5)	

**Table 4:** Survival outcome after AFR implantation.

	Death (*N* = 3)	Survivor (*N* = 37)	*p*-Value
Baseline anatomy			
Congenital heart disease	1 (33.3)	25 (67.6)	*0*.*2*
Non-congenital heart disease	2 (66.7)	12 (32.4)	
Previous surgery			
Yes	3 (100.0)	19 (51.4)	*0*.*1*
No	0 (0.0)	18 (48.6)	
Indication for AFR implantation			
ECMO	2 (66.7)	2 (5.4)	*0*.*01*
Fenestrated Fontan desaturation	0 (0.0)	5 (13.5)	
Fontan failure	1 (33.3)	3 (8.1)	
Right ventricle hypertension	0 (0.0)	11 (29.7)	
Left heart failure	0 (0.0)	16 (43.2)	
Baseline NYHA class			
I–II	0 (0.0)	15 (40.5)	*0*.*1*
III–IV	3 (100.0)	21 (56.8)	
Missing	0 (0.0)	1 (2.7)	
Venous access			
Femoral	3 (100.0)	33 (89.2)	*0*.*5*
Other	0 (0.0)	4 (10.8)	
Access to LA			
Fontan fenestration	1 (33.3)	5 (13.5)	*0*.*5*
Atrial communication	1 (33.3)	8 (21.6)	
Needle	1 (33.3)	24 (64.9)	
Balloon predilation			
Yes	2 (66.7)	24 (64.9)	*0*.*9*
No	1 (33.3)	13 (35.1)	
AFR used			
=4 mm	0 (0.0)	13 (35.1)	*0*.*2*
>4 mm	3 (100.0)	24 (64.9)	
Median of length of hospital stay (in days)	20 (16–24)	5 (1–8)	*0*.*2*

At follow-up, 20 patients improved their NYHA class, 12 patients did not change their NYHA class, and only one patient with idiopathic pulmonary hypertension worsened his NYHA class.

## Discussion

This is the largest series of CHD patients or children with pulmonary hypertension or cardiomyopathy in whom an AFR device was implanted. This device was effective in creating a stable fenestration at different levels, with a low complication rate and an encouraging improvement in functional capacity in the short-term follow-up. The AFR device has proved to be very versatile, both in patients with CHD and children with cardiomyopathies or structurally normal hearts. Most of the patients of this multicenter case series had a CHD (65.0%) with the majority being affected by left heart lesions varying from critical aortic stenosis with fibroelastosis to Shone-like complex to HLHS. Four patients had an ASD and pulmonary hypertension, one tetralogy of Fallot (ToF), one pulmonary atresia with ventricular septal defect (VSD) and major aorto-pulmonary collateral arteries (MAPCAs), and finally a patient with hemitruncus arteriosus. Among non-CHD patients, nine children had cardiomyopathies (six dilated, two restrictive, and one hypertrophic) and five presented with idiopathic pulmonary hypertension not responsive to medications and a structurally normal heart.

Despite the extreme variety and complexity of baseline anatomies, often modified by multiple surgical and percutaneous interventions, the AFR device was implanted following five main indications. In 40.0% of these patients, the AFR device was implanted for left heart failure. This population was composed of patients with congenital left ventricle inflow or outflow obstruction often undergoing multiple surgeries or children affected by dilated or restrictive cardiomyopathy. The preliminary experience of AFR implantation in patients with restrictive cardiomyopathy, as described for the first time in humans by Hansmann et al., has great potential in children with limited treatment options, and can be considered a bridge to heart transplantation or destination therapy in this particular setting (13). The rationale for implanting an AFR device in patients with dilated and restrictive cardiomyopathies, left atrial hypertension, and severe LV diastolic dysfunction is to unload the left cavities creating a left-to-right shunt, alleviate lung congestion, and improve subendocardial perfusion with a decrease in myocardial oxygen consumption (18). In particular, for three children with dilated cardiomyopathy and two with restrictive physiology and severe increase in LV end-diastolic pressure and LA pressure, the device was implanted to create a left-to-right shunt and unload the left atrium. Included in the series is a 15-year-old with complex CHD in natural history, with complete mixing, ductal-dependent systemic circulation, and combined pulmonary hypertension (PH), where the AFR device proved effective in reducing left atrial pressure, alleviating symptoms of pulmonary hypertension.

In a quarter of the cases, an atrial communication was created to unload the right heart cavities, at the price of a potential decrease in oxygen saturation, in the presence of severe pulmonary hypertension unresponsive to standard drug treatment. The intent was to maintain stroke volume during an acute pulmonary hypertension crisis that dramatically reduces transpulmonary blood flow with significant dilation of the right ventricle, which compresses the left ventricle, reducing the cardiac output and causing syncope (3–5, 19). The promising results of AFR in adults and children with advanced pulmonary arterial hypertension were shown by Sivakumar et al., who demonstrated the benefits after AFR implantation in terms of cardiac output, systemic oxygen transport, and symptoms. Indeed, over the one year follow-up, there was no recurrence of syncope, with a significant change in NYHA class and 6-min walk distance (4). However, the AFR does not pressure-unload the RV in systole and thus the AFR in severe pulmonary arterial hypertension (PAH) may prevent recurrent syncope and sudden death in PH crisis, without preventing progressive right heart failure and death in severe PAH.

**Figure 1:**
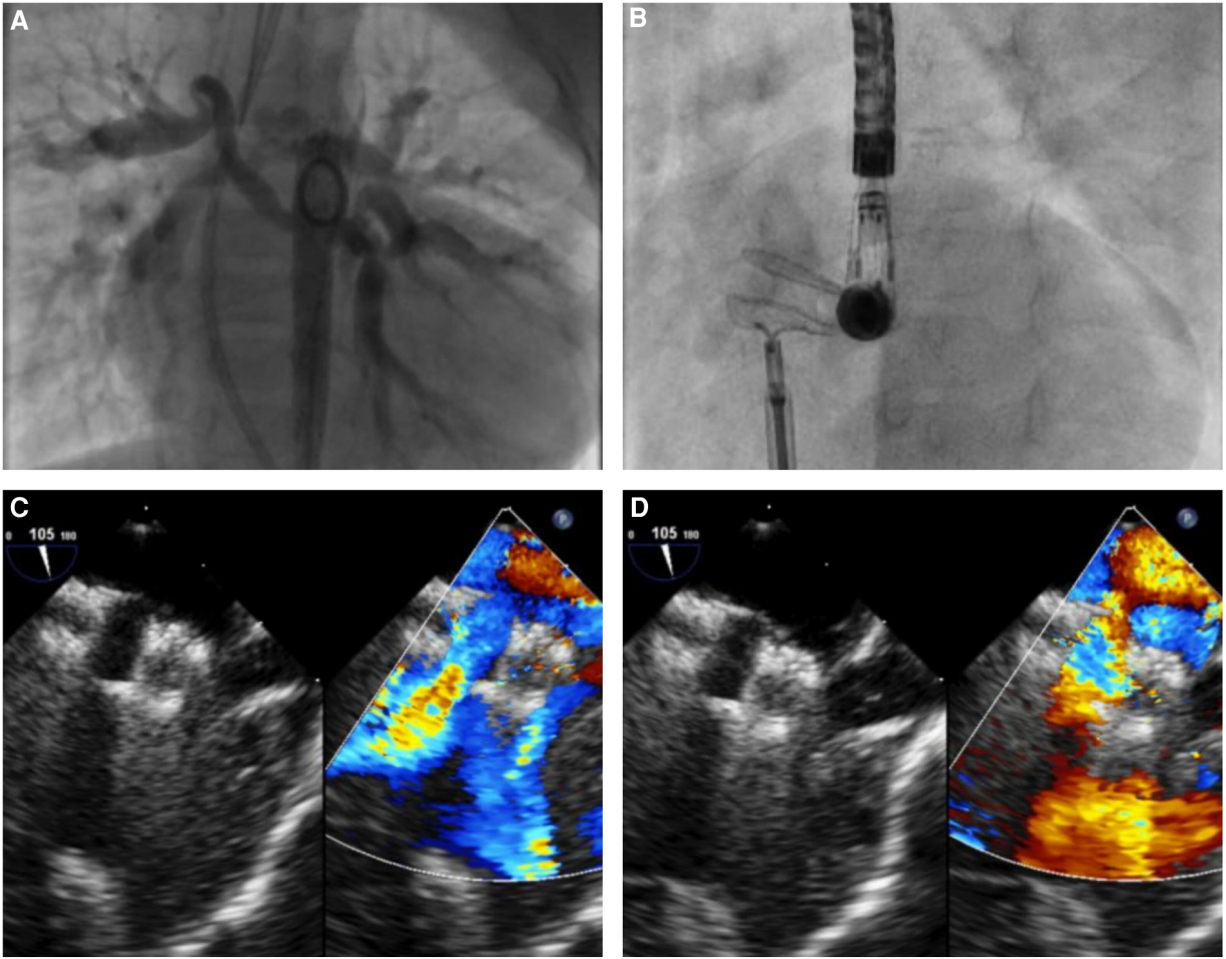
Atrial flow regulator implantation in a 22-month-old child with pulmonary atresia, restrictive ventricular septal defect, absent central pulmonary arteries, major aorto-pulmonary collaterals arteries (MAPCAs), and a hypertensive/hypertrophied right ventricle. (**A**) Aortography showing multiple MAPCAs arising from the descending aorta. (**B**) Successful implantation of an 8-mm AFR in the interatrial septum. (**C,D**) Unrestrictive bidirectional shunt across the AFR.

This category also included four adults with a secundum ASD and severe pulmonary hypertension unresponsive to standard drug treatment in whom the two largest devices, 8 and 10 mm, were used to enlarge the existing fenestration. Finally, in a 22-month-old child with pulmonary atresia with VSD and MAPCAs in native history, the AFR device was implanted in an existent small atrial communication to unload the hypertensive right cavities (Figure 1).

In the context of Fontan palliation, the implantation of an AFR was deemed to reduce the failing Fontan pressure or to reduce the size of an existing fenestration. In the patients with failing Fontan presenting with ascites, protein losing enteropathy, and/or plastic bronchitis, the creation of a right-to-left shunt at the level of the conduit allowed a reduction in the Fontan pressures at the expense of a predictable systemic desaturation (8, 9). The choice to implant an AFR device in the Fontan conduit in this setting appears to be a valid alternative to the use of a blade/balloon septostomy, stent placement, or Amplatzer-fenestrated ASD device, which presents a rate of spontaneous reocclusion of 63% with fenestrations of about 5 mm (20). In five cases, a small AFR device was implanted in patients with a Fontan circulation and severe desaturation caused by a conduit fenestration larger than 4 mm. The AFR implantation allowed the reduction of systemic cyanosis with negligible increase in pressure in the Fontan system (10, 11). A special category is that of patients with end-stage cardiomyopathy on ECMO. After the first description of AFR implantation in a child with end-stage heart failure on venoarterial ECMO (16), in this series the AFR was used in three patients with dilated cardiomyopathy and one with end-stage hypertrophic cardiomyopathy on ECMO with diastolic dysfunction and high filling pressure to unload the left cavities by creating a regulated atrial shunt. In two patients on ECMO this procedure was essential to tackle acute pulmonary edema secondary to left ventricular afterload increase and to bridge the child to heart transplant. Half of the patients on EMCO died after AFR implantation, due to sepsis and brain hemorrhage, associating this indication more with worse survival outcomes in comparison to the others.

**Figure 2:**
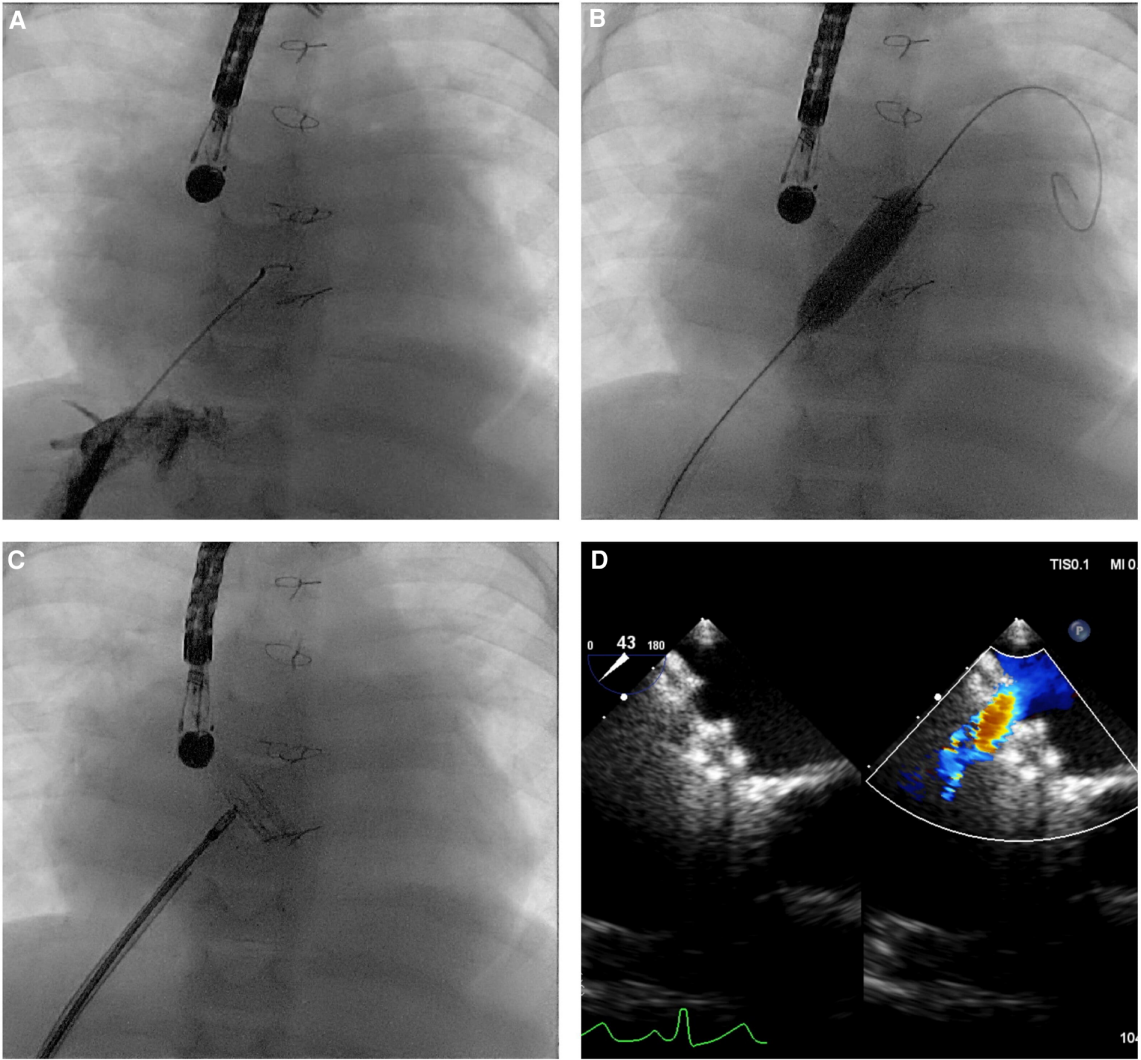
Atrial flow regulator implantation through transhepatic access in a 12-kg child with critical aortic stenosis after multiple interventions, restrictive physiology of both ventricles, and postcapillary pulmonary hypertension, on waiting list for heart transplantation. (**A**) Hepatic vein position was confirmed with injection of a small amount of contrast under fluoroscopy. (**B**) A 12-mm Powerflex balloon was inflated to predilate the intertribal septum. (**C,D**) A 6-mm AFR was implanted with unrestrictive left-to-right shunt on transesophageal echocardiography.

**Figure 3:**
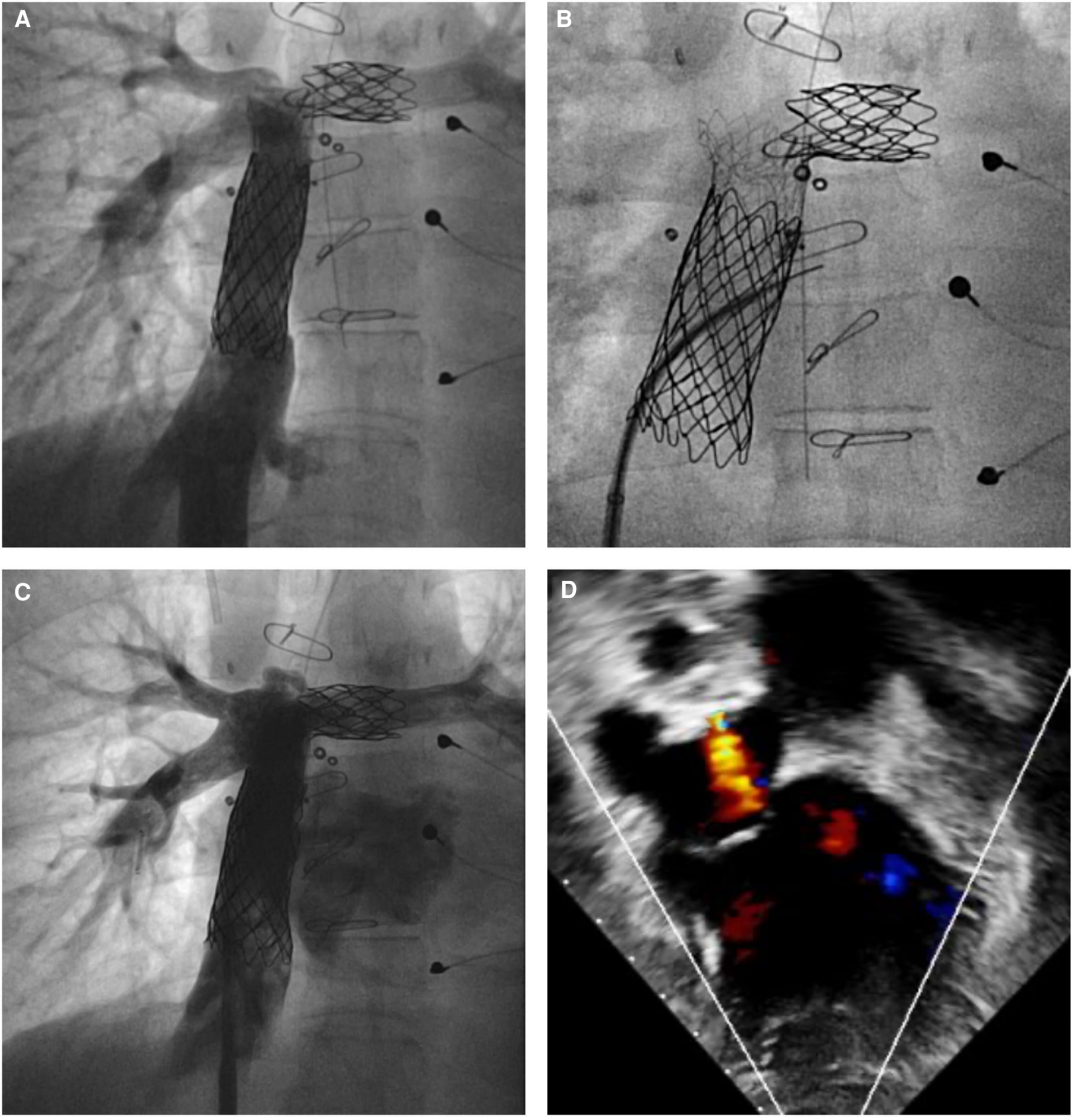
Atrial flow regulator implantation in a patient with a failing Fontan circulation. (**A,B**) Previously stented extracardiac Fontan conduit was perforated with a Brockenbrough needle associated with an electrocautery and the hard tip of a 0.014″ coronary wire. (**C,D**) A 8-mm atrial flow regulator was implanted with unrestrictive right to left shunt on transesophageal echocardiography.

The implantation of the AFR device proved to be procedurally safe with a success rate of 100%. Furthermore, the use of a relatively low delivery profile has allowed the implantation of this device in small children, especially because the AFR can be delivered through sheaths that are 1–2 Fr smaller than the ones recommended by the manufacturer (7). The median weight at the procedure in this series was 17 kg and 25% of the patients weighed ≤10 kg, confirming the safety data reported in low-weight children by Bautista et al. (7). The AFR implantation technique is relatively easy and similar to percutaneous secundum ASD closure. The procedure was generally performed under general anesthesia with ultrasound-guided femoral access. However, in the absence of femoral vascular access, it was possible to implant the device in the interatrial septum through a transhepatic access or via the right subclavian vein in a Fontan conduit. In the two transhepatic cases, a short sheath 3–4 Fr larger than the required Occlutech delivery system was used to avoid friction in the hepatic vein and liver parenchyma (Figure 2). Access to the left atrium was obtained in most cases with the help of a transeptal needle used in 22 cases to puncture the atrial septum and in three cases in a Fontan conduit. In one case, a previously stented Fontan conduit was perforated with the association of electrocautery to the Brockenbrough needle and the hard tip of a coronary wire (Figure 3). In the remaining cases, the needle was not necessary and the device was implanted through a pre-existing fenestration. As previously described, it is very often useful to predilate the created fenestration with a balloon (21). In our series, predilation was performed in 65.0% of cases with the use of intermediate-high pressure balloons with a diameter of about 4–8 mm larger than the final AFR device deployed. In some cases, a cutting balloon was used both at the level of the atrial septum and the Fontan conduit. When the fenestration was already present, it was generally not predilated with a balloon before AFR implantation.

The choice of the device remains the most challenging aspect, as there are no universal selection criteria according to the patient's weight, underlying anatomy, or indication. Assessment on a case-by-case basis is mandatory to determine the appropriate size of the fenestration. In this case series, the choice was reasonably based on patient weight and age, with 4-mm devices implanted in smaller patients (median age 33 months, IQR: 28–55) compared with 10-mm ones implanted in older patients (median age 160 months, IQR: 98–312). Moreover, a trend toward the two larger devices emerged in adult patients with ASD and pulmonary hypertension and toward the 4 and 6 mm in patients with desaturated Fontan. In patients with dilated and restrictive cardiomyopathies, a smaller balloon size/AFR fenestration ratio of 1:3 was considered to avoid unrestrictive shunts. Indeed, in this particular setting, it is essential to create a calibrated shunt that reduces left atrium volume overload and postcapillary pulmonary hypertension with a tolerable impact on cardiac output.

The rate of implantation success was 100%, free of early complications in 95.0% of the cases. Only two procedures were complicated by early device thrombosis, probably because of aggressive intraprocedural heparinization. Both occlusions in our series occurred in Fontan patients. In one patient, a fenestration was created for early extr-cardiac Fontan failure and dilated with a 7-mm high-pressure balloon. The initially implanted 4-mm AFR partially occluded during the procedure, but it was decided to leave it in and anticoagulate the patient. There was no improvement in flow and a few weeks later it was removed and replaced with a 6-mm AFR after further dilatation of the fenestration with an 8-mm cutting balloon and a 12-mm high-pressure balloon. This new device remained patent suggesting the importance of adequate preparation/predilation of the Goretex conduit or atrial septum. The risk of device embolization is reasonably low, and predilatation reduces the compressive forces on the middle of the device and potentially decreases risk of thrombosis.

Moreover, the AFR device has proved to remain patent in the majority of cases at follow-up. Indeed, the rate of patency was 91.5% at 330 days with only one late thrombosis. In total, three 4-mm devices clotted (two early and one late thrombosis) placing the smallest device at higher risk of thrombosis. However, due to the 7.5% rate of device thrombosis, the risk of systemic embolus or stroke remains one of the main concerns especially when a right-to-left shunt is created, including failing Fontan patients, which express higher rates of thromboembolism. This risk is even higher during intervention on a clotted AFR such as balloon angioplasty or removal of a clotted device.

The data from this AFR study are very valuable given the previous different techniques described to create interatrial or intrabaffle fenestrations (20, 22). We speculated that this result is due to the inability of balloon atrial septostomy to create and maintain a stable fenestration and the higher thromboembolic risk of a stent protruding on both sides of the atrial septum or baffle.

Most of the patients were discharged without complications (92.5%) after spending a median of 5 days in hospital.

One patient experienced severe brain hemorrhage on ECMO and another had a cardiac arrest and cerebral ischemia a few hours after a hybrid procedure. These events were probably related to the ECMO run and the cardiac arrest, despite the potential for embolic events in the setting of the AFR device.

Interestingly, at a mean follow-up of 330 days, 97.4% of the discharged patients were alive. Only a child died of sepsis in the local hospital more than 3 months after the procedure and all but one device maintained their patency at last follow-up.

A further encouraging aspect is that in 97.0% of patients in whom the NYHA functional class was evaluated before and after the implantation of AFR devices, a clinical improvement or non-worsening was noted.

In conclusion, AFR implantation in patients with congenital heart diseases and children with severe pulmonary hypertension or cardiomyopathy is promising and at a short-term follow-up seems to have beneficial effects. The AFR has the potential to provide benefits in terms of symptoms and survival to a variety of patients with limited treatment options and indeterminate prognosis. However, despite these promising results, large multicenter prospective registries and trials are required to confirm the efficacy and safety of this device in children and patients with CHD.

## Limitations

This retrospective study carries some limitations being a retrospective study with a relatively small sample. Furthermore, the study population is highly variable and heterogeneous, making the comparison of the different subgroups challenging. Indeed, the presence of 10 different centers made the population variable in terms of patient selection, procedural technique, and postprocedural management, making it difficult to compare patients and related outcomes. The wide heterogeneity of indications for AFR implantations and scarcity of scientific evidence for the implantations make evaluation of outcomes even more difficult. The functional status was evaluated only by the NYHA functional class as follow-up with functional capacity tests was not standardized among different centers. Another limitation is that several hemodynamic data after AFR implantation are missing. Finally, 12 patients of this study have been already published as smaller case series as single-center experiences.

## Data Availability

The original contributions presented in the study are included in the article/Supplementary Material, further inquiries can be directed to the corresponding authors.
